# Comparative features of superior versus inferior hemisphere microvasculature dropout in open-angle glaucoma

**DOI:** 10.1007/s10384-024-01071-5

**Published:** 2024-05-30

**Authors:** Naoki Takahashi, Kazuko Omodaka, Tsutomu Kikawa, Takahiro Ninomiya, Naoki Kiyota, Satoru Tsuda, Toru Nakazawa

**Affiliations:** 1 Department of Ophthalmology, Tohoku University Graduate School of Medicine, 1-1 Seiryo-machi, Aoba-ku, Sendai, 980-8574 Miyagi Japan; 2 Research & Development Division, Topcon Corporation, Tokyo, Japan; 3 Department of Retinal Disease Control, Ophthalmology, Tohoku University Graduate School of Medicine, Sendai, Japan

**Keywords:** Microvasculature dropout, OCTA, Open-angle glaucoma, Laser speckle flowgraphy, Blood pressure

## Abstract

**Purpose:**

This study aimed to investigate differences in microvasculature dropout (MvD) between the superior and inferior hemispheres in glaucoma patients.

**Study design:**

Retrospective and cross-sectional.

**Methods:**

Fifty-eight eyes of 58 open-angle glaucoma patients (age 61.12 ± 10.19 years, mean deviation − 7.32 ± 6.36 dB) were included. MvD was detected with en face images from swept-source optical coherence tomography angiography. Blood flow at the optic nerve head was measured with laser speckle flowgraphy, represented as the mean blur rate in tissue (MBRT). Logistic and linear regression models adjusted for age, intraocular pressure, axial length, and circumpapillary retinal nerve fiber layer thickness were used to investigate the relationship between various factors and MvD angle in each hemisphere.

**Results:**

The presence of inferior MvD was related to peripapillary atrophy-β area (odds ratio = 14.10 [2.49–234.00], *P* = 0.019). Superior MvD angle was significantly related to MBRT in the superior quadrant (β = −0.31 [− 0.60 – −0.02], *P* = 0.037). Inferior MvD angle was significantly related to peripapillary atrophy-β area (β = 0.49 [0.21–0.77], *P* = 0.001).

**Conclusions:**

Only superior MvD demonstrated a significant relationship with reduced ocular blood flow. In contrast, inferior MvD was associated with mechanical stress. These findings may suggest a potential difference in pathophysiology between superior and inferior MvD.

## Introduction

Glaucoma is the second most common cause of blindness worldwide [1, 2]. In general, it is considered that high intraocular pressure (IOP) leads to damage to the lamina cribrosa where the optic nerve runs through it. Therefore, therapies aimed at reducing IOP, whether through medication or surgical intervention, are considered effective treatments to prevent glaucoma progression [3, 4]. However, despite successful IOP reduction, many patients, particularly those with normal-tension glaucoma, still demonstrate visual field progression [5]. Additionally, risk factors other than elevated IOP have been identified [6]. One important non-IOP risk factor is low ocular blood flow [7]. We reported that low ocular blood flow precedes the reduction of circumpapillary retinal nerve fiber layer thickness (cpRNFLT) in some glaucoma cases [8]. Therefore, it is also important to evaluate ocular blood flow in glaucoma patients.

The advent of swept-source optical coherence tomography (SS-OCT) enabled more precise investigation in deeper layers of the retina and choroid. SS-OCT employs a relatively long wavelength (1050 nm), enabling deep penetration, and produces high-resolution images of these deeper layers, including the choroidal layer [9, 10]. SS-OCT also has faster scanning speed and allows patients to maintain good fixation due to its low glare, providing highly detailed images. Thus, SS-OCT has the advantage of producing clear capillary images of these deeper layers.

Recently, choroidal microvasculature dropout (MvD), which can be observed in the peripapillary atrophy-β (PPAβ) zone, has been reported as a characteristic marker associated with glaucoma progression [11]. MvD represents localized vascular dropout associated with retinal nerve fiber layer defects (RNFLDs) [11], disc hemorrhage [12], and central visual field dysfunction [13]. Studies using indocyanine green angiography show that the short posterior ciliary artery supplies the lamina cribrosa through capillaries in the PPAβ [13]. Thus, MvD indicates localized vascular dysfunction, which reduces the blood supply in the lamina cribrosa [14, 15] and might be a key to revealing the relationship between ocular blood flow and glaucoma progression.

Differences in initial glaucomatous damage between the superior and inferior hemispheres have been documented. It is reported that IOP fluctuations are associated with greater damage to the inferior optic disc [16]. In contrast, our previous findings indicate that in older patients, a higher pulse rate is associated with an increased risk of reduced ocular blood flow, which precedes cpRNFL thinning in the superior and temporal quadrants [8]. We also reported that reduced optic nerve head blood flow in the superior quadrant at baseline was significantly associated with subsequent inferior visual field progression [17]. Based on these findings, we hypothesized that the mechanism of glaucoma progression may vary depending on the specific quadrant. In the present study, we aimed to investigate whether superior and inferior MvD were associated with ocular/systemic parameters. Our results highlight the disparities between superior and inferior MvD and the implications of these disparities.

## Materials and methods

### Subjects

This study included 58 eyes of 58 patients with primary open-angle glaucoma (POAG) or normal-tension glaucoma (NTG). The initial diagnosis was made by a glaucoma specialist (T.N.) between January 2016 and September 2020 at Tohoku University Hospital. Glaucoma was defined in this study by the presence of an abnormal glaucomatous optic disc (with diffuse or focal thinning of the neuroretinal rim) and a corresponding glaucomatous visual field defect, defined according to the Anderson-Patella criteria [18] by the presence of one or more of the following: (1) a cluster of three points with reduced sensitivity at a probability of < 5% on the pattern deviation map in at least one hemifield (including ≥ 1 point at a probability of < 1% or a cluster of two points at a probability of < 1%), (2) glaucomatous hemifield test results outside the normal limits, and (3) a pattern standard deviation beyond 95% of normal limits, as confirmed in at least two reliable examinations. A peak IOP of 21 mmHg or less without any glaucoma medication was diagnosed as NTG, and a peak IOP of more than 21 mmHg was diagnosed as POAG. The number of hypertension and diabetes drugs was self-reported for this study. Eyes with cataract (a lens nucleus grade 3 or more by Emery-Little classification), vitreoretinal or optic nerve diseases other than glaucoma were excluded from the current study. All patients had PPAβ, atrophy of the retinal pigment epithelium and choriocapillaris [19], and RNFLDs with clear boundaries in en face images of 12 × 9 mm OCT wide scans. When both eyes of a patient were eligible, the eye with the wider RNFLD angle was included. This study adhered to the tenets of the Declaration of Helsinki, and the protocols were approved by the Clinical Research Ethics Committee of the Tohoku University Graduate School of Medicine (study 2021-1-430).

### Measurement of variables

**Fig. 1:**
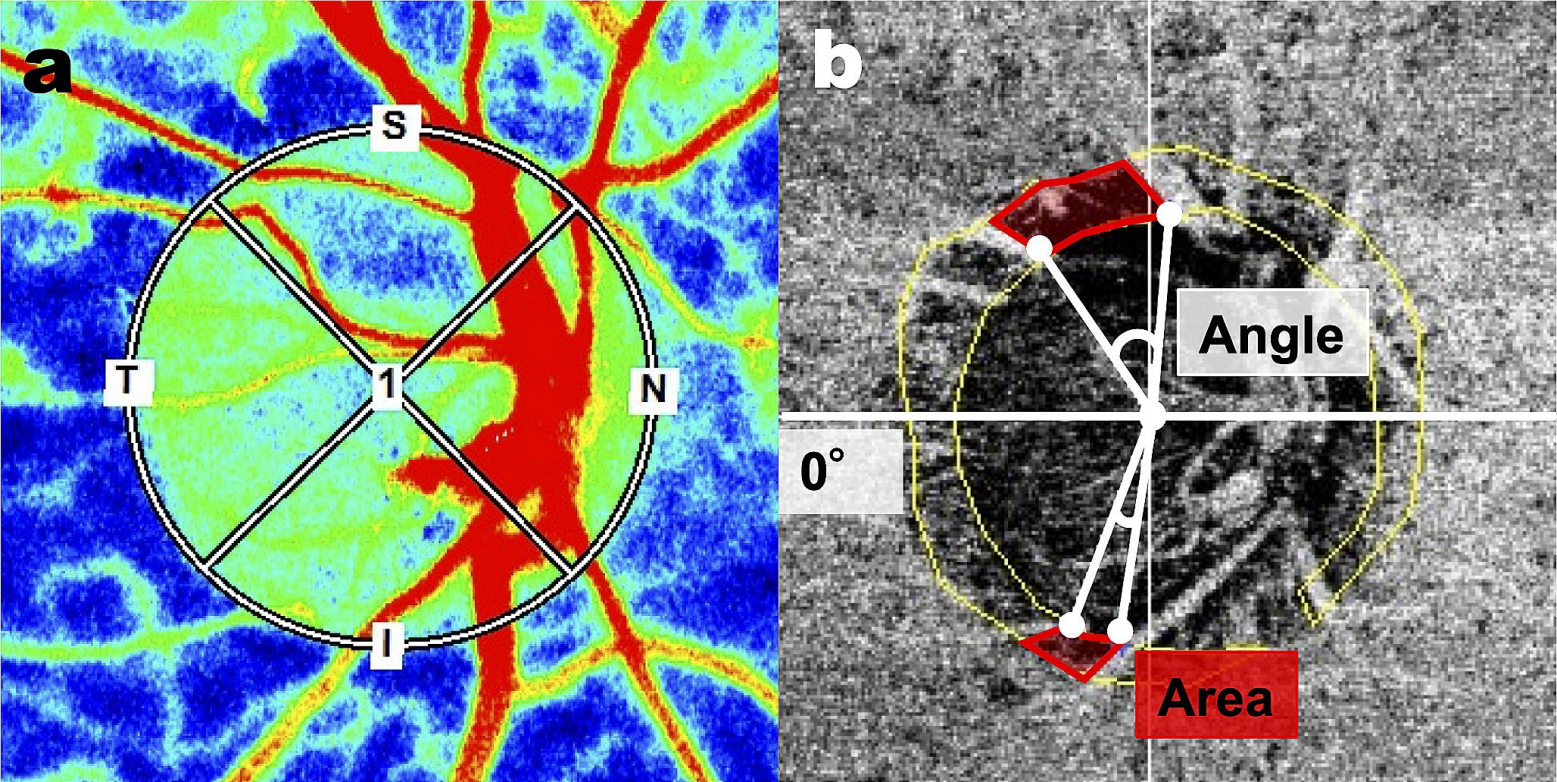
Representative laser speckle flowgraphy measurements and an en face image obtained with optical coherence tomography angiography. (**a**) We measured the blood flow within the optic nerve head (ONH) in the tissue area (white ellipse), as well as in the separate quadrants. (**b**) The yellow line illustrates the margin of the peripapillary atrophy-β (PPAβ) zone. Microvasculature dropout (MvD) was detected when it was more than two times greater than the width of visible capillaries in the PPAβ zone (red line). The MvD area was measured within the red line. The MvD angle was determined by identifying the two points where its extreme borders met the ONH border as angular circumferential margins and drawing two lines connecting the ONH center (white line). To determine the positional coordinates of MvD, we set the temporal side as 0 degrees

Best-corrected visual acuity (BCVA) was measured with a standard Japanese decimal visual acuity chart and converted to the logarithm of the minimum angle of resolution (logMAR). IOP was measured with Goldmann applanation tonometry. Axial length was measured with ocular biometry (OA-2000, Tomey Corporation). Central corneal thickness (CCT) was measured with anterior-segment OCT (CASIA2, Tomey Corporation). Optic nerve head (ONH) blood flow was assessed with laser speckle flowgraphy (LSFG; LSFG-NAVI, Softcare Co, Ltd.), which measures mean blur rate (MBR) in arbitrary units (AU). In the current study, we used MBR in the tissue area (MBRT) to assess capillary blood flow; we measured overall MBRT as well as MBRT in the superior, temporal, and inferior quadrants (Fig. 1a). Mean deviation (MD) was measured with the Humphrey field analyzer (HFA; Carl Zeiss Meditec) with the Swedish interactive threshold algorithm (SITA)–standard strategy of the 24 − 2 program. Only reliably measured MD values were used (< 20% fixation errors, < 15% false-positive results, and < 33% false-negative results). All the examination data were obtained within a two-month period, without interrupting the use of medication for glaucoma.

Clinical parameters for each patient were recorded, including age, sex, and body mass index (BMI). BMI was calculated by the following formula: weight (kg) / height^2^ (m^2^). Blood pressure (BP) and pulse rate (PR) were conventionally measured in the brachial artery at the height of the heart (HBP-1300, Omron Colin Co.). Mean arterial pressure (MAP) and ocular perfusion pressure (OPP) were calculated with the following formulas, respectively: MAP = diastolic BP + 1/3 (systolic BP − diastolic BP); OPP = 2/3MAP − IOP.

### OCT examination

CpRNFL thickness (cpRNFLT) was measured in a 12 × 9 mm wide scan with swept-source OCT (SS-OCT; DRI OCT Triton, Topcon Corp.). Retinal nerve fiber layer defect (RNFLD) angle was measured in en face images according to a method described in previous reports. Briefly, the RNFLD angle was measured as the intersection between the RNFLD and a circle centered on the disc with a radius half the distance between the disc and the fovea, using software we have developed [20, 21].

### Measurement of MvD and PPAβ area

In this study, 4.5 × 4.5 mm OCT angiography (OCTA) images centered on the ONH were acquired with SS-OCTA (DRI OCT Triton, Topcon Corp.). Only good-quality images with image quality > 30 were included [22]. The PPAβ border was marked in projection images using Label Studio, an open-source data labeling tool (Heartex Inc.). We overlaid the PPAβ margin on the en face image, which represented a layer starting at 130 μm below the internal limiting membrane (ILM) and reaching 390 μm below Bruch’s membrane; this layer includes the full thickness of the choroid and the scleral flange [12, 23]. MvD was evaluated on the overlaid en face image using Label Studio and was defined as local, sectorial capillary dropout without any visible microvascular network; this capillary dropout was more than two times greater than the width of the visible capillaries [11, 24]. Based on the labeled data, we calculated the area of both the PPAβ zone and MvD. MvD angle was determined by identifying the two points where its extreme borders met the ONH border as angular circumferential margins and drawing two lines connecting the ONH center to these margins (Fig. 1b). To determine the positional coordinates of MvD, we set the temporal side at 0 degrees, with the superior side at 90 degrees (moving clockwise), the nasal side at 180 degrees, and the inferior side at 270 degrees.

Both the PPAβ zone and MvD margins were evaluated by two glaucoma specialists (N.T. and K.O.), who were masked to all clinical data, including cpRNFL and visual field sensitivity. Any uncertainty was resolved by another glaucoma specialist (T.N.). If an eye had more than one MvD, all the MvDs were calculated separately then added for the superior and inferior hemispheres. If an MvD extended across both the superior and inferior hemispheres, the MvD angle was measured in each hemisphere separately.

### Statistical analysis

Correlations were calculated with the Pearson correlation coefficient. Logistic regression analysis was performed to examine the presence of MvD in the superior and inferior hemispheres. Linear regression models were used to evaluate the magnitude of MvD. All models were adjusted for potential confounding factors, including age, IOP, axial length, and cpRNFLT [22, 25]. Analyses were performed using JMP software (version 17.0.0; SAS Institute Japan Inc.) or R (version 4.1.2; R Foundation for Statistical Computing). P values < 0.05 were considered statistically significant. No adjustments were made for multiple comparisons, and 95% confidence intervals (CIs) are provided for all results to enable comparison of parameters.

## Results

**Fig. 2:**
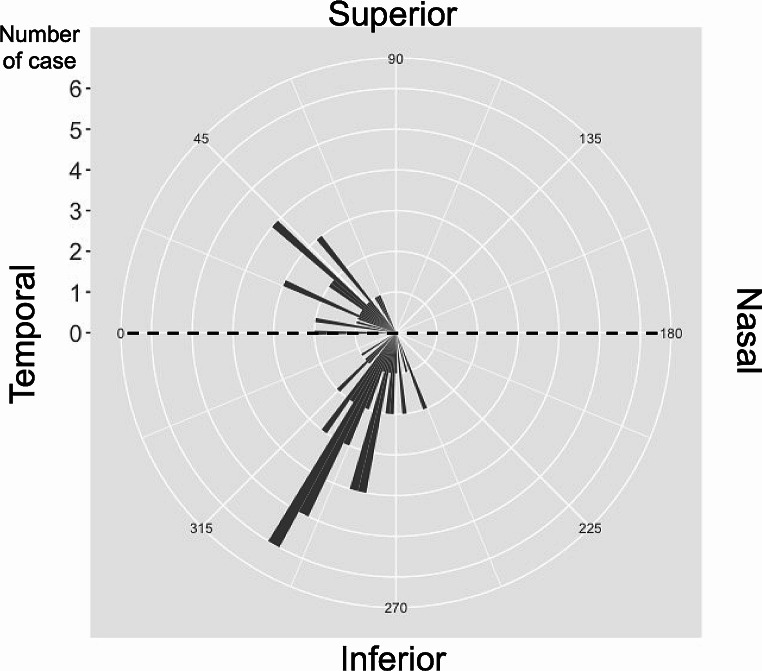
Rose diagram illustrating the positional coordinates of microvasculature dropout (MvD). We set the temporal side as 0 degrees, with the superior at 90 degrees (moving clockwise), the nasal at 180 degrees, and the inferior at 270 degrees

Table 1 shows the characteristics of the participants in this study. The average age was 61.12 ± 10.19 years, axial length was 25.06 ± 1.34 mm, MD was − 7.32 ± 6.36 dB, PPAβ area was 1.24 ± 0.98 mm^2^, MvD angle was 32.95 ± 20.12, and MvD area was 0.21 ± 0.23 mm^2^. Table 2 shows the correlation between the MvD angle/area and the various ocular/systemic parameters. MvD angle showed significant correlation with glaucoma severity (cpRNFLT: β = − 0.48 [− 0.71 – −0.24], *P* < 0.001; MD: β = − 0.55 [− 0.77 – −0.32], *P* < 0.001; RNFLD angle: β = 0.68 [0.49–0.88], *P* < 0.001). Both axial length and PPAβ area were significantly correlated with MvD area (β = 0.35 [0.10–0.60], *P* = 0.008, β = 0.75 [0.58–0.93], *P* < 0.001, respectively); however, glaucoma severity was not. Consequently, we used MvD angle in subsequent analyses. Table 3 shows the results of multivariable regression models adjusted for confounding factors (i.e., age, IOP, axial length, and cpRNFLT). The models show that MvD angle was significantly related to systolic BP, MAP, OPP, and PPAβ area (β = −0.29 [− 0.55 – −0.04], *P* = 0.024; β = −0.26 [− 0.50 – −0.01], *P* = 0.040; β = −0.25 [− 0.50 – −0.01], *P* = 0.040; β = 0.41 [0.15–0.67], *P* = 0.003, respectively). Figure 2 presents a rose diagram illustrating the locations of MvD. As shown in the figure, MvD was most frequently located inferotemporally and next most frequently located superotemporally. We did not observe any MvDs that extended across the superior and inferior regions in this study.

**Table 1:** Characteristics of the participants

Characteristic	*N* = 58
Age, y	61.12 (10.19)
Sex, n (%)	
Female	31 (53%)
Male	27 (47%)
Glaucoma type, n (%)	
NTG	46 (79%)
POAG	12 (21%)
BMI, kg/m^2^	22.52 (3.11)
Systolic BP, mmHg	126.82 (18.50)
Diastolic BP, mmHg	77.84 (14.44)
MAP, mmHg	94.17 (14.92)
PR, /min	72.27 (11.75)
BCVA, logMAR	−0.05 (0.15)
IOP, mmHg	13.51 (2.54)
CCT, µm	513.57 (34.32)
AL, mm	25.06 (1.34)
MD, dB	−7.32 (6.36)
MBRT, AU	10.17 (2.19)
CpRNFLT, µm	74.99 (11.95)
RNFLD, degree	
Total	43.71 (28.76)
Superior	18.47 (20.49)
Inferior	25.24 (17.96)
PPA area, mm^2^	1.24 (0.98)
MvD angle, degree	
Total	32.95 (20.12)
Superior	11.14 (12.64)
Inferior	21.81 (17.26)
MvD area, mm^2^	
Total	0.21 (0.23)
Superior	0.05 (0.08)
Inferior	0.06 (0.09)
Medications, n (%)	
Prostaglandin analogs	34 (94%)
β-antagonists	26 (72%)
Carbonic anhydrase inhibitors	25 (69%)
α2-stimulators	22 (61%)
Rho-kinase inhibitors	12 (33%)
Hypertension, n (%)	13 (22%)
Diabetes, n (%)	4 (7%)

AL, axial length; AU, arbitrary unit; BCVA, best-corrected visual acuity; BMI, body mass index; BP, blood pressure; CCT, central corneal thickness; cpRNFLT, circumpapillary retinal nerve fiber layer thickness; IOP, intraocular pressure; MAP, mean arterial pressure; MBRT, mean blur rate in tissue; MD, mean deviation; MvD, microvasculature dropout; NTG, normal-tension glaucoma; POAG, primary open-angle glaucoma; PPA, peripapillary atrophy; PR, pulse rate; RNFLD, retinal nerve fiber layer defect.

**Table 2:** Correlation between MvD angle/area and ocular/systemic parameters

		MvD angle				MvD area		
		β	95% CI	P		β	95% CI	P
Age		−0.06	−0.33–0.21	0.659		−0.02	−0.28–0.25	0.909
BMI		−0.13	−0.40–0.14	0.345		−0.25	−0.50–0.01	0.056
Systolic BP		−0.36	−0.61 – −0.11	0.006		−0.23	−0.50–0.03	0.079
Diastolic BP		−0.31	−0.57 – −0.06	0.016		−0.21	−0.47–0.05	0.117
MAP		−0.35	−0.60 – −0.10	0.007		−0.23	−0.50–0.03	0.082
PR		−0.16	−0.43–0.11	0.248		−0.13	−0.39–0.14	0.340
IOP		−0.15	−0.41–0.12	0.262		−0.15	−0.41–0.12	0.277
CCT		−0.17	−0.43–0.09	0.205		−0.09	−0.35–0.18	0.513
AL		0.14	−0.13–0.40	0.297		0.35	0.10–0.60	0.008
OPP		−0.32	−0.57 – −0.07	0.013		−0.20	−0.47–0.06	0.132
MBRT		−0.25	−0.52–0.01	0.060		−0.03	−0.30–0.24	0.828
PPA area		0.27	0.01–0.53	0.043		0.75	0.58–0.93	< 0.001
CpRNFLT		−0.48	−0.71 – −0.24	< 0.001		−0.08	−0.34–0.19	0.562
MD		−0.55	−0.77 – −0.32	< 0.001		−0.25	−0.51–0.01	0.059
RNFLD angle		0.68	0.49–0.88	< 0.001		0.31	0.06–0.56	0.018
β: standard regression coefficient								

AL, axial length; BMI, body mass index; BP, blood pressure; CCT, central corneal thickness; CI, confidence interval; cpRNFLT, circumpapillary retinal nerve fiber layer thickness; IOP, intraocular pressure; MAP, mean arterial pressure; MBRT, mean blur rate in tissue; MD, mean deviation; MvD, microvasculature dropout; OPP, ocular perfusion pressure; PPA, peripapillary atrophy; PR, pulse rate; RNFLD, retinal nerve fiber layer defect.

**Table 3:** Multivariable regression models for MvD angle/area and ocular/systemic parameters

		MvD angle				MvD area			
		β	95% CI	P		β	95% CI	P	
BMI		−0.09	−0.35–0.18	0.523		−0.23	−0.49–0.04	0.089	
Systolic BP		−0.29	−0.55 – −0.04	0.024		−0.17	−0.44–0.11	0.233	
Diastolic BP		−0.22	−0.46–0.03	0.080		−0.17	−0.43–0.09	0.195	
MAP		−0.26	−0.50 – −0.01	0.040		−0.18	−0.44–0.09	0.184	
PR		−0.06	−0.32–0.20	0.651		−0.10	−0.36–0.16	0.449	
OPP		−0.25	−0.50 – −0.01	0.040		−0.18	−0.44–0.09	0.184	
MBRT		0.01	−0.28–0.31	0.937		0.13	−0.17–0.43	0.394	
PPA area		0.41	0.15–0.67	0.003		0.79	0.59–0.98	< 0.001	
β: standard partial regression coefficient, adjusted for age, IOP, AL, and cpRNFLT									

AL, axial length; BMI, body mass index; BP, blood pressure; CI, confidence interval; cpRNFLT, circumpapillary retinal nerve fiber layer thickness; IOP, intraocular pressure; MAP, mean arterial pressure; MBRT, mean blur rate in tissue; MvD, microvasculature dropout; OPP, ocular perfusion pressure; PPA, peripapillary atrophy; PR, pulse rate

Next, we performed a logistic regression analysis to investigate which factors were related to superior or inferior MvD (Table 3). The analysis showed that superior MvD was not significantly, but was likely related to, low systolic BP, low MAP, and low OPP (OR = 0.96 [0.92–1.00], *P* = 0.062; OR = 0.96 [0.91–1.00], *P* = 0.092; OR = 0.94 [0.87–1.00], *P* = 0.092, respectively), after adjusting for age, IOP, AL, and cpRNFLT. On the other hand, inferior MvD was significantly related to high PPAβ area (OR = 14.10 [2.49–234.00], *P* = 0.019) in the multivariable model.

Our investigation of the relationship between MvD angle and other factors in both the superior and inferior hemispheres had the following results. For superior MvD, a significant relationship was found with MBRT (β = −0.32 [− 0.58 – −0.07], *P* = 0.015), particularly superior MBRT (β = −0.42 [− 0.66 – −0.17], *P* = 0.001) and temporal MBRT (β = −0.30 [− 0.56 – −0.04], *P* = 0.023); for inferior MvD, there was a significant relationship with PPAβ area (β = 0.32 [0.07–0.58], *P* = 0.014). These relationships remained significant even after adjusting for age, IOP, AL, and cpRNFLT: superior MvD with superior MBRT (β = −0.31 [− 0.60 – −0.02], *P* = 0.037) and inferior MvD with PPAβ area (β = 0.49 [0.21–0.77], *P* = 0.001).

## Discussion

In this study, we categorized MvD based on its location in the superior or inferior hemispheres to explore potential differences in pathology between MvD in these two locations. Our findings indicate a significant association between lower MvD and PPAβ after adjusting for potential confounding factors (as shown in Table 4). In terms of MvD angle, a notable relationship was identified between superior MvD and superior MBRT. Conversely, while inferior MvD angle was significantly related with PPAβ area, no significant relationships were observed between inferior MvD and any of the other circulation parameters, as detailed in Table 5. Overall, these results imply that superior MvD is uniquely associated with reduced ocular circulation.

**Table 4:** Logistic regression analysis of factors associated with superior and inferior MvD

		Superior MvD								Inferior MvD						
		Univariable				Multivariable				Univariable				Multivariable		
		OR	95% CI	P		OR	95% CI	P		OR	95% CI	P		OR	95% CI	P
Age, per 1y		1.00	0.95–1.05	0.928		–	–	–		1.03	0.97–1.10	0.317		–	–	–
BMI, per 1		0.96	0.80–1.14	0.635		0.97	0.79–1.19	0.785		0.82	0.64–1.01	0.077		0.78	0.58–1.02	0.084
Systolic BP, per 1 mmHg		0.96	0.93–0.99	0.023		0.96	0.92–1.00	0.062		0.98	0.94–1.01	0.175		0.97	0.92–1.01	0.123
Diastolic BP, per 1mmHg		0.96	0.92–1.00	0.054		0.97	0.92–1.01	0.147		0.96	0.91–1.00	0.076		0.96	0.91–1.01	0.113
MAP, per 1 mmHg		0.95	0.91–0.99	0.030		0.96	0.91–1.00	0.092		0.96	0.92–1.01	0.090		0.96	0.91–1.01	0.104
PR, per 1/min		1.00	0.95–1.05	0.946		1.01	0.96–1.08	0.582		0.98	0.93–1.04	0.508		0.98	0.92–1.05	0.639
IOP, per 1 mmHg		0.90	0.72–1.10	0.314		–	–	–		0.80	0.59–1.04	0.106		–	–	–
CCT, per 1 μm		0.99	0.97–1.00	0.121		0.99	0.97–1.01	0.331		1.00	0.98–1.02	0.948		1.01	0.99–1.04	0.250
AL, per 1 mm		1.31	0.88–2.00	0.195		–	–	–		0.68	0.38–1.14	0.170		–	–	–
OPP, per 1 mmHg		0.94	0.87–0.99	0.048		0.94	0.87–1.00	0.092		0.96	0.89–1.02	0.167		0.94	0.87–1.01	0.104
MBRT, per 1 AU		0.75	0.56–0.97	0.038		0.87	0.62–1.18	0.383		0.98	0.73–1.33	0.902		1.48	0.93–2.60	0.129
PPA area, per 1 mm^2^		1.27	0.74–2.38	0.404		1.41	0.72–2.99	0.327		3.21	1.06–16.65	0.100		14.10	2.49–234.00	0.019

Multivariable models were adjusted for age, IOP, AL, and cpRNFLT

AL, axial length; AU, arbitrary unit; BMI, body mass index; BP, blood pressure; CCT, central corneal thickness; CI, confidence interval; cpRNFLT, circumpapillary retinal nerve fiber layer thickness; IOP, intraocular pressure; MAP, mean arterial pressure; MBRT, mean blur rate in tissue; MvD, microvasculature dropout; OPP, ocular perfusion pressure; OR, odds ratio; PPA, peripapillary atrophy; PR, pulse rate

**Table 5:** Association between superior/inferior MvD angle and other parameters

		Superior MvD angle								Inferior MvD angle							
		Univariable				Multivariable				Univariable				Multivariable			
		β	95% CI	P		β	95% CI	P		β	95% CI	P		β	95% CI	P	
Age		−0.04	−0.31–0.22	0.741		–	–	–		−0.04	−0.30–0.23	0.786		–	–	–	
BMI		−0.03	−0.31–0.25	0.836		0.03	−0.25–0.31	0.833		−0.13	−0.41–0.15	0.351		−0.12	−0.42–0.18	0.419	
Systolic BP		−0.25	−0.51–0.01	0.060		−0.17	−0.44–0.11	0.230		−0.23	−0.49–0.03	0.080		−0.22	−0.51–0.07	0.134	
Diastolic BP		−0.22	−0.48–0.05	0.106		−0.12	−0.38–0.14	0.347		−0.21	−0.47–0.06	0.120		−0.16	−0.44–0.12	0.246	
MAP		−0.24	−0.51–0.02	0.068		−0.15	−0.41–0.12	0.272		−0.23	−0.49–0.03	0.084		−0.19	−0.47–0.09	0.173	
PR		0.01	−0.26–0.29	0.916		0.10	−0.17–0.37	0.459		−0.20	−0.47–0.08	0.158		−0.14	−0.43–0.15	0.327	
IOP		−0.17	−0.43–0.09	0.199		–	–	–		−0.05	−0.32–0.22	0.714		–	–	–	
CCT		−0.22	−0.48–0.05	0.103		−0.12	−0.40 –0.15	0.375		−0.04	−0.31–0.23	0.773		0.06	−0.25–0.35	0.716	
AL		0.16	−0.10–0.43	0.225		–	–	–		0.04	−0.22–0.31	0.744		–	–	–	
OPP		−0.21	−0.47–0.06	0.124		−0.14	−0.41–0.12	0.272		−0.23	−0.49–0.04	0.089		−0.19	−0.47–0.09	0.173	
PPA area		−0.01	−0.28–0.25	0.911		−0.02	−0.31–0.27	0.885		0.32	0.07–0.58	0.014		0.49	0.21–0.77	0.001	
MBRT_All		−0.32	−0.58 – −0.07	0.015		−0.17	−0.47–0.13	0.261		−0.06	−0.33–0.21	0.667		0.14	−0.19–0.46	0.397	
MBRT_Superior		−0.42	−0.66 – −0.17	0.001		−0.31	−0.60 – −0.02	0.037		0.04	−0.23–0.31	0.774		0.27	−0.05–0.58	0.096	
MBRT_Temporal		−0.30	−0.56 – −0.04	0.023		−0.12	−0.42–0.18	0.419		−0.07	−0.35–0.20	0.585		0.08	−0.24–0.41	0.611	
MBRT_Inferior		−0.11	−0.38–0.16	0.414		0.03	−0.25–0.31	0.829		−0.15	−0.42–0.12	0.258		−0.02	−0.32–0.28	0.886	
Multivariable models were adjusted for age, IOP, AL, and cpRNFLT; β: standard (partial) regression coefficient																	

AL, axial length; BMI, body mass index; BP, blood pressure; CCT, central corneal thickness; CI, confidence interval; cpRNFLT, circumpapillary retinal nerve fiber layer thickness; IOP, intraocular pressure; MAP, mean arterial pressure; MBRT, mean blur rate in tissue; MvD, microvasculature dropout; OPP, ocular perfusion pressure; PPA, peripapillary atrophy; PR, pulse rate.

First, we measured both the MvD angle and area. As evident from the results in Table 2, the MvD angle, when compared to the MvD area, had a stronger correlation with more parameters of glaucoma severity. Consequently, we chose the MvD angle for our subsequent investigations. On the other hand, the MvD area demonstrated a strong correlation with axial length and PPAβ area, suggesting that the MvD area measurements were predominantly influenced by these anatomical parameters.

Reduced ocular circulation, indicative of low BP and OPP, was significantly associated with a wider MvD angle (Table 3). This is consistent with Lee et al.’s identification of low MAP and OPP as risk factors for MvD [23]. While our multivariable logistic models did not demonstrate significant relationships between the superior/inferior hemispheres and ocular circulation parameters (Table 4), the borderline p-values suggest that further investigation is warranted. Although low BP can be a risk factor of MvD, hypertension is concurrently a significant risk factor for glaucoma [26]. Elevated blood pressure not only affects IOP but, due to microvascular damage from systemic hypertension, is a risk factor for POAG [27]. Therefore, both high and low blood pressure are reported to be harmful in glaucoma patients [28], highlighting the need for optimal blood pressure regulation.

In the linear regression models examining the association between MvD angle in the superior/inferior hemispheres and other parameters, a significant association was observed only between the superior hemisphere and reduced superior MBRT. A previous report demonstrated that MvD in the peripapillary region is frequently adjacent to MvD in the optic disc [29], which aligns with the findings of this study. However, the specific reasons for the difference in ocular circulation between the superior and inferior hemispheres remain unclear. Previous reports show that inferior MBRT also has a significant association with the corresponding (i.e., superior) visual field [17]. Therefore, it is unlikely that measuring inferior MBRT would be challenging. On the other hand, inferior MvD has shown a strong association with PPAβ, suggesting that the expansion of MvD due to disc rotation may be a physical cause, which could explain the lack of association with inferior MBRT. Our findings of an association in the superior hemisphere are supported by several past studies. Suzuki et al. found that normal-tension glaucoma patients who had signs of ischemic changes in brain MRI predominantly had inferior pericentral visual field disturbances [30]. Tomita et al. observed that blood flow in the superior retina decreased significantly compared to the inferior retina following postural changes in healthy subjects [31]. Similarly, Kiyota et al. indicate that older open-angle glaucoma patients with disturbances in the superior and temporal cpRNFL had an increased risk of ocular blood flow reduction preceding cpRNFL loss [8]. The specific mechanism rendering the superior hemisphere more vulnerable to reduced blood flow remains unclear, but these studies reinforce our observations.

The presence and magnitude of inferior MvD were significantly associated with the PPAβ area (Tables 4 and 5). PPAβ enlargement has been shown to be associated with axial elongation caused by myopia [32]. This elongation may increase the distance between the Zinn-Haller arterial circle and the optic disc, potentially leading to blood vessel ruptures [33] and the consequent development of inferior MvD. While our findings did not show a relationship between inferior MvD and IOP, other studies suggest such a relationship. For example, Midgett et al. found pronounced distortion in the inferior quadrant when increased pressure was applied to the lamina cribrosa in human donor eyes [34]. Thus, a possible association between inferior MvD and mechanical stresses, such as axial elongation or elevated IOP, could exist, but this question warrants further investigation.

There were several limitations in this study. (1) The study had a limited number of participants who were all of a single ethnicity. Being a single-center and cross-sectional study further limits its generalizability. (2) By its very design, MvD can only be detected within the PPA zone, making it a challenge to assess deep vessels outside this area. This limitation is particularly pronounced in glaucoma patients with smaller PPA zones. (3) The cross-sectional nature of the study means that we cannot determine if the development of superior MvD is a result of reduced ocular blood flow or a precursor to it. A longitudinal study would provide clarity on the causality of this relationship. (4) Poor OCTA image quality could hinder the detection of MvD. To ensure accuracy, we excluded images with an image quality score below 30. (5) Our method for generating en face images from OCTA was consistent, drawing from established practices in prior studies [12, 23]. However, using a slab reference 130 μm downward from the ILM might occasionally overlook MvD or misjudge its size. This calls for a potential re-evaluation of the slab creation methodology. (6) The study’s retrospective nature and the limited number of cases without inferior MvD might mean potential underlying factors associated with the formation of inferior MvD went unnoticed. In light of these considerations, future studies might benefit from larger, more diverse samples, more refined imaging techniques, and a longitudinal design. (7) In this study, we did not perform adjustment for multiple comparisons. This is particularly relevant in the analysis of quartiles of MBRT, where there may have been a need for multiple comparisons. As Rothman has noted [35], while corrections for multiple comparisons reduce the risk of type I errors, they simultaneously increase the risk of type II errors. Our primary objective was to report new findings that could be clinically useful. Therefore, to facilitate comparison of parameters, we have provided the 95% confidence intervals for all β coefficients [36].

In conclusion, our findings suggest distinct mechanisms for superior and inferior MvD development. While superior MvD appears to be influenced by reduced blood flow, the size of the PPAβ area may be pivotal for inferior MvD. Such observations underscore the importance of stratifying glaucoma based on its underlying pathophysiology. This study builds on our previous work, in which we found that various risk factors, such as decreased blood flow and aging, were associated with visual acuity decline in glaucoma patients [37], and suggests that understanding the disease’s pathophysiology in a more segmented manner offers promise. This could very well lay the groundwork for a tailored approach to treating glaucoma, moving toward a more personalized paradigm in medicine.
